# System analysis based on the cuproptosis-related genes identifies LIPT1 as a novel therapy target for liver hepatocellular carcinoma

**DOI:** 10.1186/s12967-022-03630-1

**Published:** 2022-10-04

**Authors:** Cheng Yan, Yandie Niu, Liukai Ma, Lifang Tian, Jiahao Ma

**Affiliations:** School of Pharmacy, Key Laboratory of Nano-Carbon Modified Film Technology of Henan Province, Diagnostic Laboratory of Animal Diseases, Xinxiang University, Xinxiang, Henan China

**Keywords:** Liver hepatocellular carcinoma, Cuproptosis, Prognostic model, LIPT1

## Abstract

**Background:**

Liver hepatocellular carcinoma (LIHC) ranks sixth among the most common types of cancer with a high mortality rate. Cuproptosis is a newly discovered type of cell death in tumor, which is characterized by accumulation of intracellular copper leading to the aggregation of mitochondrial lipoproteins and destabilization of proteins. Thus, understanding the exact effects of cuproptosis-related genes in LIHC and determining their prognosticvalue is critical. However, the prognostic model of LIHC based on cuproptosis-related genes has not been reported.

**Methods:**

Firstly, we downloaded transcriptome data and clinical information of LIHC patients from TCGA and GEO (GSE76427), respectively. We then extracted the expression of cuproptosis-related genes and established a prognostic model by lasso cox regression analysis. Afterwards, the prediction performance of the model was evaluated by Kaplan–Meier survival analysis and receiver operating characteristic curve (ROC). Then, the prognostic model and the expression levels of the three genes were validated using the dataset from GEO. Subsequently, we divided LIHC patients into two subtypes by non-negative matrix factorization (NMF) classification and performed survival analysis. We constructed a Sankey plot linking different subtypes and prognostic models. Next, we calculate the drug sensitivity of each sample from patients in the high-risk group and low-risk group by the R package pRRophetic. Finally, we verified the function of LIPT1 in LIHC.

**Results:**

Using lasso cox regression analysis, we developed a prognostic risk model based on three cuproptosis-related genes (GCSH, LIPT1 and CDKN2A). Both in the training and in the test sets, the overall survival (OS) of LIHC patients in the low-risk group was significantly longer than that in the high-risk group. By performing NMF cluster, we identified two molecular subtypes of LIHC (C1 and C2), with C1 subtype having significantly longer OS and PFS than C2 subtype. The ROC analysis indicated that our model had a precisely predictive capacity for patients with LIHC. The multivariate Cox regression analysis indicated that the risk score is an independent predictor. Subsequently, we identified 71 compounds with IC50 values that differed between the high-risk and low-risk groups. Finally, we determined that knockdown of LIPT1 gene expression inhibited proliferation and invasion of hepatoma cells.

**Conclusion:**

In this study, we developed a novel prognostic model for hepatocellular carcinoma based on cuproptosis-related genes that can effectively predict the prognosis of LIHC patients. The model may be helpful for clinicians to make clinical decisions for patients with LIHC and provide valuable insights for individualized treatment. Two distinct subtypes of LIHC were identified based on cuproptosis-related genes, with different prognosis and immune characteristics. In addition, we verified that LIPT1 may promote proliferation, invasion and migration of LIHC cells. LIPT1 might be a new potential target for therapy of LIHC.

**Supplementary Information:**

The online version contains supplementary material available at 10.1186/s12967-022-03630-1.

## Introduction

Liver hepatocellular carcinoma (LIHC) is one of the most common malignancies worldwide, ranking as the sixth most common type of cancer globally [1]. According to the World Health Organization, liver cancer will kill more than 1 million people by 2030 [2]. The LIHC has a poor prognosis and high mortality rate worldwide, with only 18% of patients surviving 5 years, which is lower than bladder cancer (77.1%), renal pelvis cancer (74.8%) and myeloma (52.2%) [3].

At present, patients with hepatocellular carcinoma are mainly treated with liver transplantation, hepatectomy, radiofrequency ablation, transcatheter arterial chemoembolization (TACE) and radioembolization [4–6]. However, due to LIHC has an insidious onset, rapid progression, and low early diagnosis rate, most cases of LIHC tend to be diagnosed at an advanced stage and miss the best opportunity for treatment. Therefore, it is essential to identify novel biomarkers that simultaneously serve as prognostic predictive markers and therapeutic targets for LIHC.

Copper plays an important role in cells as a catalytic cofactor for essential enzymes involved in energy conversion, oxygen transport, and regulation of oxidative metabolism in cells [7]. The concentration of copper in cells is regulated by metabolic demands and changes in the cellular environment, and too little or too much can cause significant damage to cells [8]. Imbalance of copper metabolism can seriously affect the development of the central nervous system and have an impact on the normal metabolism of the liver [9]. Copper ions will regulate cell death in a distinct manner when the intracellular concentration of copper ions reaches a certain level by targeting lipoylated TCA cycle proteins [10]. A recent study found that accumulation of intracellular copper triggers aggregation of mitochondrial lipoproteins and destabilization of proteins, leading to a unique type of cell death called cuproptosis [11, 12]. The Cuprotosis gene affects the process of tumor initiation, invasion, and metastasis in a manner similar to the ferroptosis and pyroptosis genes. Cuprotosis is closely related to cancer progression and is expected to be a novel therapeutic target to specifically kill cancer cells [13, 14]. However, the prognostic model of LIHC based on cuproptosis-related genes has not been reported.

In this study, we developed a prognostic risk model based on three cuproptosis-related genes by performing LASSO cox regression and multivariate cox regression analysis. Both in the training and in the test sets, the OS of LIHC patients in the low-risk group was significantly longer than that in the high-risk group. The Kaplan–Meier curve and the ROC curve were performed to estimate the sensitivity and specificity of the prognostic signature. By performing NMF cluster, we identified two molecular subtypes of LIHC (C1 and C2), with C1 subtype having significantly longer OS and PFS than C2 subtype. Then, we performed drug sensitivity analysis, which might provide a novel reference index for determining prognosis risk and selecting treatment strategies for LIHC patients. Finally, we validated the function of LIPT1 in LIHC by knocking down its expression level, LIPT1 may provide a potential therapeutic target.

## Methods

### Data acquisition

The TCGA (The Cancer Genome Atlas) database was created by the National Cancer Institute and contains genomic, transcriptomic, proteomic, and methylation data for 20,000 primary cancers (http://cancergenome.nih.gov/). From TGCA, we collected 424 LIHC patients transcriptomic data and corresponding clinical information. From GEO (GSE76247), we collected 167 LIHC patients transcriptomic data and corresponding clinical information (https://www.ncbi.nlm.nih.gov/).

### Expression of extracted cuproptosis-related genes

Firstly, we extracted the expression of cuproptosis-related genes in the expression matrix of the training set. We then extracted the expression of cuproptosis-related genes in the test set expression matrix similarly and corrected the extracted genes from the training set and the test set.

### The construction and validation of a prognostic model based on prognostic cuproptosis-related genes

Firstly, we performed Lasso cox regression analysis to avoiding overfitting of prognostic risk model variables and built prognostic models. The risk score was calculated using the formula as follows:

$$ {\text{risk}}\,{\text{score}}\,{ = }\,{\text{Coef}}_{{{1}\,}} \, \times \,\,{\text{Gene}}\,{\text{expression}}_{{1}} \,{ + }\,{\text{Coef}}_{{2}} \, \times \,{\text{Gene}}\,{\text{expression}}_{{2}} \,{ + }\, \cdots \,\,{\text{Coef}}_{{\text{n}}} \, \times \,{\text{Gene}}\,{\text{expression}} $$

The Coef represents each gene’s prognostic value in multivariate Cox regression analysis. A gene expression value represents the expression value of a corresponding prognostic cuproptosis gene. The test set was used to validate the prognostic risk score model built from the training set. R “survival” package is a tool for statistical analysis and visualization of survival data and is widely used in scientific research work [15, 16]. Using the “survival” package in R (version 4.1.2), we calculated the overall survival analysis and plotted the Kaplan–Meier survival curves. Chi-square tests were applied to the calculation of p values [17]. ROC curves were drawn using the R package “survivalROC” to verify the accuracy of the predictive model.

### NMF classification of molecular subgroups

Firstly, Spearman correlation was performed to analyze the relationship between the expression level of cuproptosis-related genes and prognostic value. Subsequently, we performed non-negative matrix factorization (NMF) clustering analysis to develop the molecular subtypes based on the expression profiles of 3 cuproptosis-related modeling genes. For the NMF method, the standard “brunet” option was selected and 10 iterations were performed. The number of clusters was set to range from 2 to 10, and the average profile width of the common membership matrix was determined by the R package “NMF”, with the minimum membership of each subclass set to 10. The optimal number of clusters was determined by co-occurrence, dispersion and contour indexes, and the optimal number of clusters selected was 2. Using the “survival” package in R, we analyzed OS and PFS for subtypes and plotted Kaplan–Meier survival curves. The “GSVA” package was used for ssGSEA analysis.

### Construction of the nomogram for patients with LIHC

The nomogram containing the clinical characteristics was established to predict individual survival probability by the “rms” package of R software [18]. To assess the consistency between actual survival time and predicted prognosis in the nomogram, calibration curves for predicting 1-, 3-, and 5 year survival rate were plotted.

### Clinical relevance analysis

In order to investigate whether there are differences between the clinical characteristics of LIHC patients in high- and low-risk, we first drew a heatmap. By using the Chi-square test, we performed a correlation analysis on each significant clinical feature.

### Analysis of immune cell infiltration

The Cell-type Identification by Estimating Relative Subsets of RNA Transcripts (CIBERSORT) method is a general way to measure cell fractions based on the gene expression profiles (GEPs), which can accurately estimate the immune component of tumor biopsies [19]. using the CIBERSORT deconvolution method, we calculated the composition of 22 tumor-infiltrating immune cell in each tumor sample, and then performed the Wilcoxon test to compare the difference of immune cells infirtraion between high-risk and low-risk group [20]. The level of statistical significance was set at P < 0.05.

### Enrichment analysis of KEGG and GO pathways

The gene ontology (GO) and Kyoto Encyclopedia of Genes and Genomes (KEGG) enrichment analysis of the differentially expressed genes between high and low risk groups were performed to find the enriched biological pathways and functions related to the cuproptosis-related genes by clusterprofiler R package [21, 22]. The enriched results for GO and KEGG analysis were visualized by “ggplot2” package.

### Calculating sensitivity score of potential durgs

pRRophetic is an R package that uses tumor gene expression levels to predict clinical chemotherapy responses [23]. The half-maximal inhibitory concentration (IC50) of compounds obtained from the Genomics of Drug Sensitivity in Cancer (GDSC) website. Using the pRRophetic package in R software, we calculate the sensitivity score of each compound for each patient in the high-risk group and low-risk group. The statistical difference was performed by Wilcox test with a P value less than 0.05 as the threshold. To visualize the conformations of drugs in 2D, PubChem online tool (https://pubchem.ncbi.nlm.nih.gov/) was used.

### Tumour mutation analysis

By using the maftool package in R software, 15 genes with the highest tumour mutation frequency (TMF) in patients with LIHC from TCGA were analysed and visualized.

Using the R “ggalluvial” package, we plotted Sankey plots for the relationship between patients in the high and low risk groups and patients with NMF subtypes.

### Cell culture

HepG2 and Hep3B cells are acquired from American Type Culture Collection. HepG2 and Hep3B cells are cultured with RPMI-1640 supplemented with 2 mM l-glutamine and 10% FBS.

### Western blotting

The LIPT1 antibody (AV48784) was obtained from Simga. The expression of LIPT1 in cells was evaluated using typical Western blotting, which actin as a loading control. Then, the protein signal was determined by ECL reagent. Subsequently, two siRNAs were applied to knock down expression level of LIPT1. The siRNA sequences were as follows: si-LIPT1-1: 5’-GGA AAU ACG UGA CAA AUU AAA-3’, si-LIPT1-2: 5’-CGU GAC AAA UUA AAU UCA AGU-3’.

### Cell viability assay

HepG2 and Hep3B cells transfected with si-LIPT1 or si-scrambled siRNA in 6-well plate. Twenty-four hours after transfection, the cells number was countered and 4000 cells were seeded into 96-well plates. The cell viability was acquired at indicated time points using the CCK8 kit.

### Clone formation tests

Cells transfected with control and siRNA were seeded into 6-well plates. After 2 weeks, colonies were stained using crystal violet.

### Edu assay

HepG2 and Hep3B cells were seeded in 24-well plates then transported with scrambled or two independent siRNA targeting LIPT1. After 48 h, cells were added with EdU and continued incubating for another 2 h. Then, the cells were fixed with a 4% paraformaldehyde solution for 30 min. The staining process was perfermed according to the manufacturer’s instructions. Images were captured using Nikon microscope and the numbers of positive cells were calculated using the imageJ software.

### Wound healing assay

The ability of cell migration was evaluated by wound healing experiment. HepG2 and Hep3B cells transfected with si-LIPT1 or si-scrambled siRNA were inoculated in 6-well plates. When the cells reach reaching a confluence of 100%. Use a 10 µL pipette to form a wound in the center of the cell monolayer, and then continue to culture in the incubator. Images were captured at 0 and 48 h after the scratch by an optical microscope. The wound area was measured by ImageJ software at indicated time points and normalized with starting time point.

### Cell invasion assay

Transwell assay was conducted to determine cell invasion. Transfected cells were collected, resuspended in serum-free RPMI-1640 medium, and cultured on Matrigel-coated upper chamber surfaces. The lower chamber was filled with FBS medium. After 24 h, the upper membrane surface was wiped with a cotton swab to remove the remaining cells. The cells adhering to the lower membrane were then fixed by using 4% paraformaldehyde and stained with crystal violet. Then, cells were photographed using a light microscope. Finally, cells were counted using ImageJ.

## Result

### The construction and internal validation of a prognostic model based on prognostic cuproptosis-related genes

**Fig. 1:**
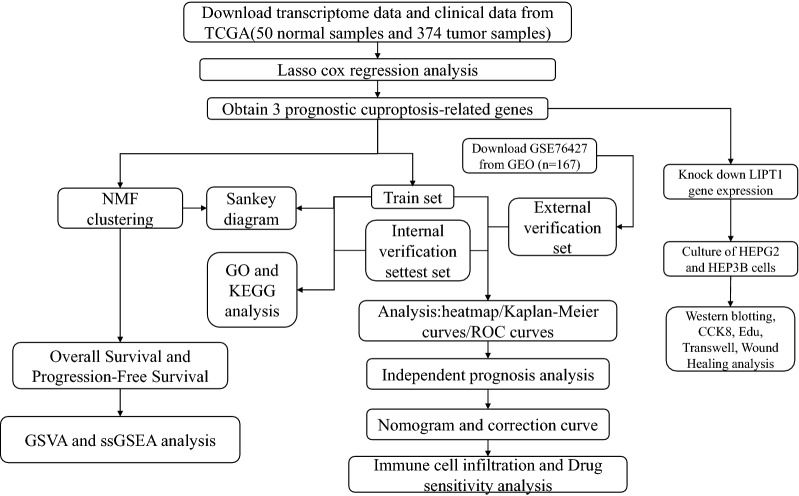
A flowchart of the major steps in this study

The flow chart shows the overall experimental design of this study (Fig. 1).

In order to construct the prognostic model and evaluate its performance, we randomly divided the TCGA data into training and internal validation set in a 1:1 ratio. We conducted the LASSO cox regression analysis to build the cuproptosis-related genes prognostic model for patients with liver hepatocellular carcinoma (Additional file 1: Figure S1A, B). The prognostic model was constructed with GCSH, LIPT1 and CDKN2A, and the risk score was calculated as follows:

$$ {\text{Risk score}}\, = \,\left( {0.{4379}0\, \times \,{\text{GCSH}}} \right)\, + \,\left( {0.{18261}\, \times \,{\text{LIPT1}}} \right)\, + \,\left( {0.0{1477}\, \times \,{\text{CDKN2A}}} \right) $$

**Fig. 2:**
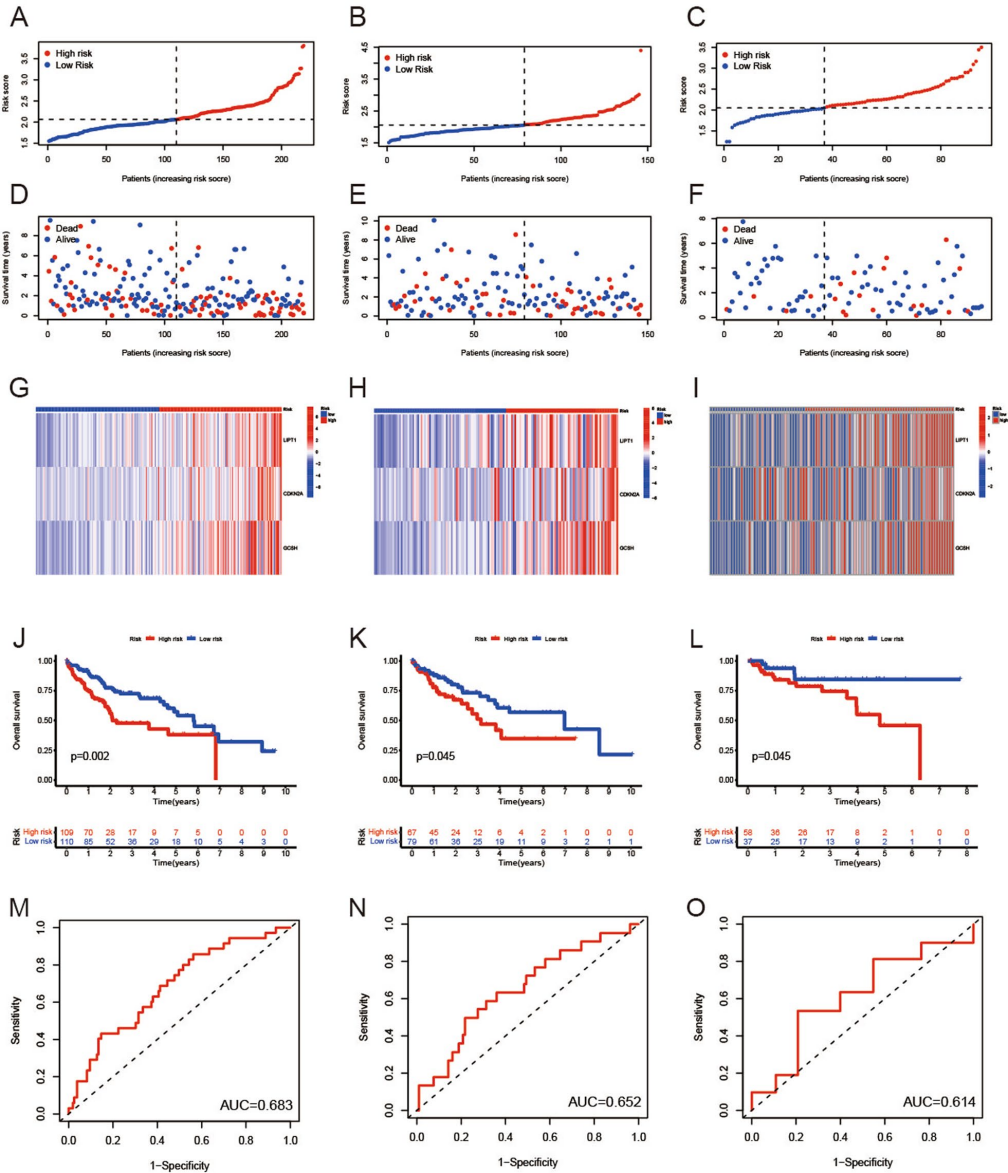
Correlation between the risk score and overall survival of LIHC patients in the training and validation set. **A** The distribution of risk scores in the training set. **B** The distribution of risk scores in the internal validation set. **C** The distribution of risk scores in the external validation set. **D** The survival status of patients in the training set. **E** The survival status of patients in the internal validation set. **F** The survival status of patients in the external validation set. **G** Heat map of 3 genes expression in the training set. **H** Heat map of 3 genes expression in the internal validation set. **I** Heat map of 3 genes expression in the external validation set. **J** Kaplan–Meier curves of survival in training set. **K** Kaplan–Meier curves of survival in internal validation set. **L** Kaplan–Meier curves of survival in external validation set. **M** Time-dependent ROC curve of the risk score model for predicting 1 years in training set. **N** Time-dependent ROC curve of the risk score model for predicting 1 years in internal validation set. **O** Time-dependent ROC curve of the risk score model for predicting 1 years in external validation set

Patients were assigned to high-risk and low-risk groups according to the median risk score (Fig. 2A, B). The prognosis of LIHC patients in the low-risk group was better than that in the high-risk group in both the training set and the internal validation set (Fig. 2D, E). The heatmap was used to visualize the expression levels of the 3 cuproptosis-related genes in the high- and low-risk group patients (Fig. 2G, H). Survival curves indicated that patients with LIHC in the low-risk group had a significantly higher survival probability compared to the patients in high-risk group (p < 0.05) (Fig. 2J, K). ROC analysis showed that the area under the curve (AUC) for 1 year OS was 0.683 for the training set and 0.652 for the internal validation set (Fig. 2M, N). Clearly, our model is helpful in predicting the outcome of LIHC patients.

### Validation of the prognostic model

In order to validate the prognostic model in external validation set, we calculate the risk score of each patient in the external validation set according to the same risk score formula we constructed. The patients in the external validation set were divided into the high-risk group and low-risk group based on the median risk score value of the training set (Fig. 2C). The survival status, and the heatmap of these 3 prognostic genes in the external validation set are shown in Fig. 2F, I. Consistent with the results of the training set, patients from the high-risk group in the external validation set showed a poorer prognosis compared to the patients from the low-risk group (Fig. 2L). In addition, ROC analysis showed an AUC of 0.614 for 1 year OS (Fig. 2O). These data suggested that our prognostic model could also accurately predict prognosis of LIHC patients from external validation set.

### Identification of independent prognostic indicator

To verify whether our prognostic model risk score could be an independent prognostic factor to predict the prognosis of patients with LIHC, we performed univariate and multivariate cox proportional hazard model in training set and test set. In the training set, the univariate and multivariate regression analysis showed that Stage and risk score were independent prognostic factors (Additional file 2: Figure S2A, B). In the test set, independent univariate regression analysis showed Stage and risk Score were independent prognostic factors (Additional file 2: Figure S2C, D). These data indicated that the signature-based risk score was an independent prognostic indicator in LIHC, which might be useful to guide clinical decision-making and diagnosis.

### Construction of nomogram and calibration curves

In order to accurately estimate survival for individual patients with LIHC, we establish a nomogram to evaluate the survival probability at 1, 3, and 5 years based on risk scores and other clinicopathological characteristics (Additional file 3: Figure S3A). Our results demonstrated that nomograms could be served as an effective tool for the prognostic evaluation of patients with LIHC. Moreover, calibration curves for OS indicated that the predicted prognosis was in good agreement with the actual mortality at 1, 3, and 5 years (Additional file 3: Figure S3B, C, D). These findings revealed that the nomogram we built could accurately assess the OS of patients with LIHC.

### Clustering of molecular subgroup

**Fig. 3:**
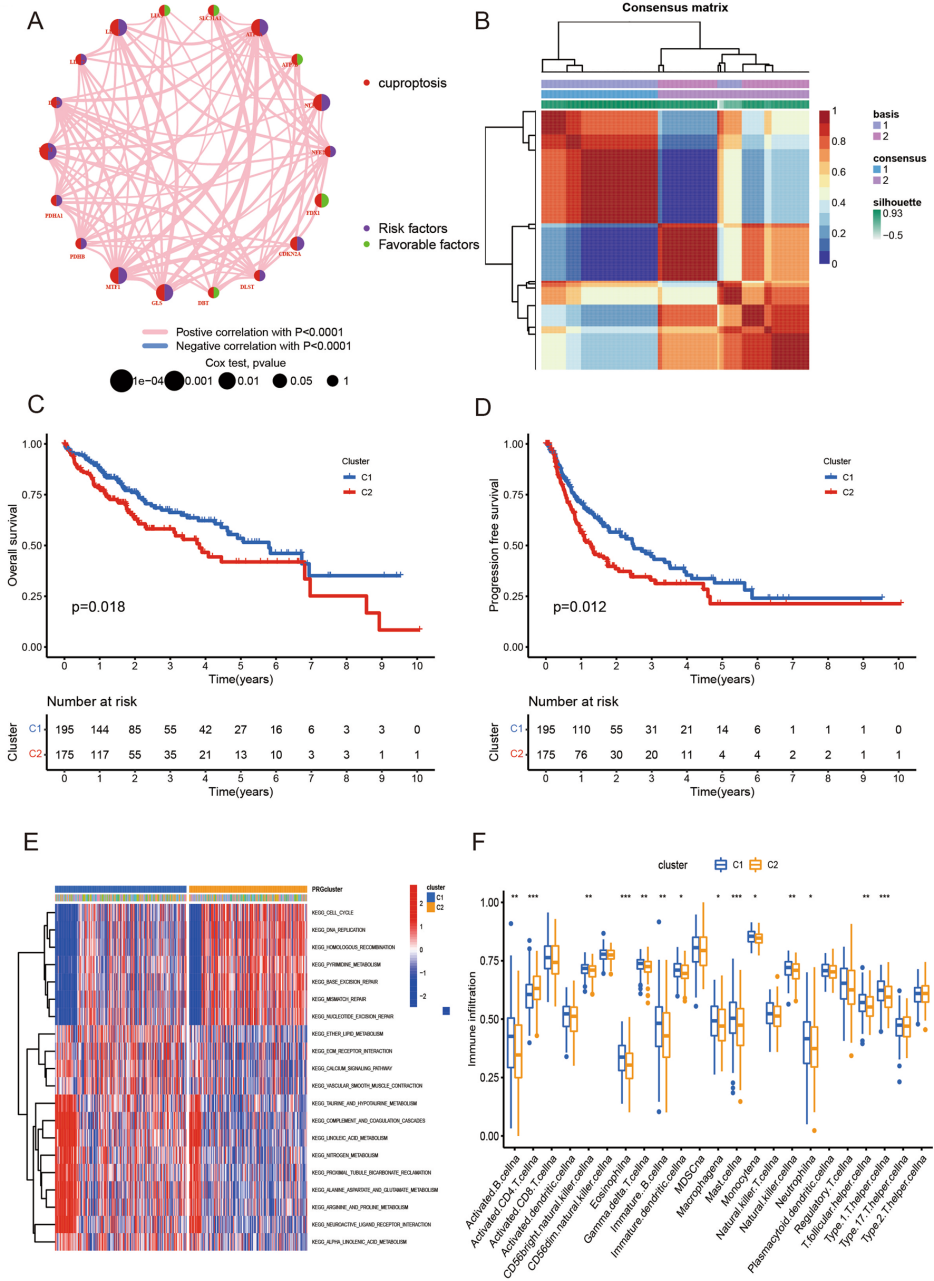
Molecular subgroups were screened by non-negative matrix factorization (NMF) clusters. **A** The network diagram illustrates the relationship between cuproptosis-related genes. Where purple represents a risk factor and green represents a favorable factor. **B** Two subgroups were identified as optimal values for consensus clustering. **C** OS survival curves for both subtypes. **D** PFS survival curves for both subtypes **E** Heatmap of GSVA analysis between the two subgroups. **F** Box plots for ssGSEA analysis between two subgroups

The network diagram illustrates the relationship between cuproptosis-related genes (Fig. 3A). We found that all the 18 cuproptosis-related genes have significant value for predicting the prognosis of LIHC patients. Among them, 13 genes are risk factors, including NFE2L2, NLRP3, ATP7A, LIPT1, LIPT2, DLD, DLAT, PDHA1, PDHB, MTF1, GLS, CDKN2A and DLST. And the others are favorable factors, including FDX1, ATP7B, SLC31A1, LIAS and DBT.

Molecular subgroups were initially classified by NMF consensus clustering on the basis of three cuproptosis-related genes that had been screened. The consensus map shows that LIHC from TCGA data were classified into two clusters (Fig. 3B). The Kaplan–Meier curves showed that either OS and RFS period of C1 was significantly longer than that of C2 with the P values being 0.018 and 0.012, respectively (Fig. 3C, D).

GSVA analysis showed that subtype C1 was significantly enriched in the cell cycle, DNA replication, homologous recombination pathway, and subtype C2 was significantly enriched in linoleic acid metabolism and nitrogen metabolism (Fig. 3E). The ssGSEA analysis showed that subtype C1 was significantly enriched in helper T cells, principal cells and eosinophils, and subtype C2 was significantly enriched in CD4 T cells (Fig. 3F).

### Clinicopathological features in the low-risk and high-risk groups

**Fig. 4:**
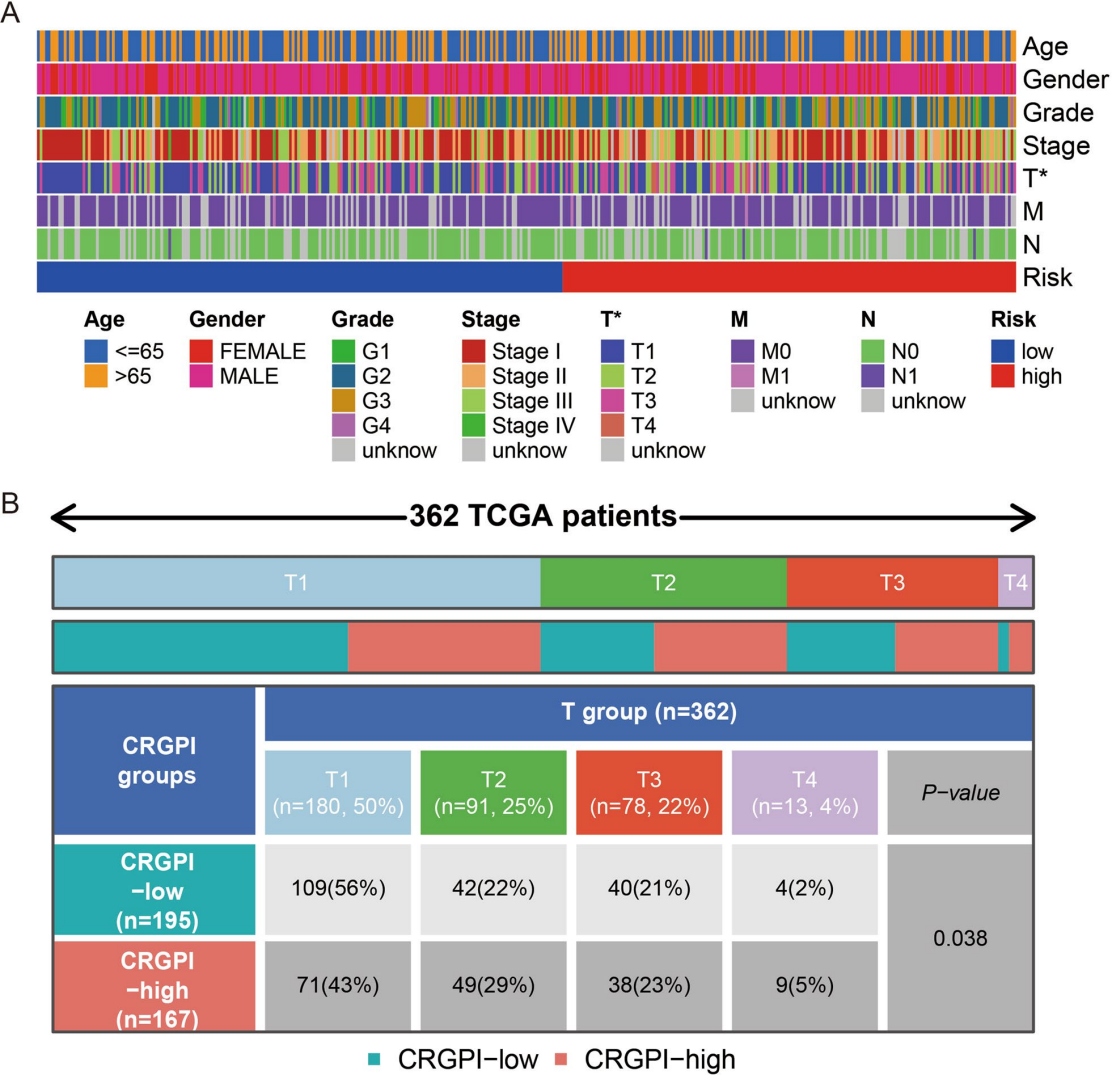
The clinical correlation analysis. **A** Clinical Correlation Analysis Heatmap. **B** T stage clinical correlation chart

In order to indicate the distribution of clinicopathological characters in the low-risk and high-risk groups, we performed correlaion analysis between the clinicopathological features and the risk signature. This heatmap is used to visualize the correlation between high and low risk groups and clinicopathologic characters (Fig. 4A). Our results indicated that the proportion of T2 and T3 patients were almost equally distributed between the two groups, but there were more T4 patients and fewer T1 patients in the CRGPI-high (cuproptosis related gene-based prognostic index) subgroup than in the CRGPI-low subgroup (Fig. 4B).

### Correlation between tumor immune cell infiltration and risk score

As tumor immune infiltration played a key role in tumorigenesis and progression, we further compared the difference of infiltration immune cells between high and low risk groups via CIBERSORT analysis. Barplot and heatmap showed the composition of 22 subpopulations of immune cells in high-risk and low-risk group (Additional file 4: Figure S4A, B). The proportion of monocytes and T cells CD4 memory resting cells was significantly higher in the low-risk group than in the high-risk group. Conversely, the proportion of macrophages M0 was lower in the low-risk group than in the high-risk group (Additional file 4: Figure S4C). In addition, survival curves showed that LIHC patients with high plasma cell infiltration rates had significantly better prognosis than those with low infiltration rates (Additional file 5: Figure S5A). Similarly, LIHC patients displaying high T cell CD8 infiltration exhibited superior OS to those displaying low CD8 + cell infiltration (Additional file 5: Figure S5B).

### Differences in expression of immune checkpoint molecules between the high-risk group and low-risk group

Comparing the immune checkpoint genes in the high-risk group to those in the low-risk group. We found that the expression of GLS, GCSH, MTF1, FDX1, NFE2L2, LIPT1, DLAT, CDKN2A and ATP7A had statistically significant differences (P < 0.001) between high-risk and low-risk groups (Additional file 6: Figure S6A).

Spearman correlation analysis showed that GLS, MTF1 was significantly positively correlated with ATP7A, and GCSH, LIPT1, and CDKN2A were significantly positively correlated with risk scores (Additional file 6: Figure S6B).

### Functional analysis of differentially expressed genes between high- and low- risk groups

**Fig. 5:**
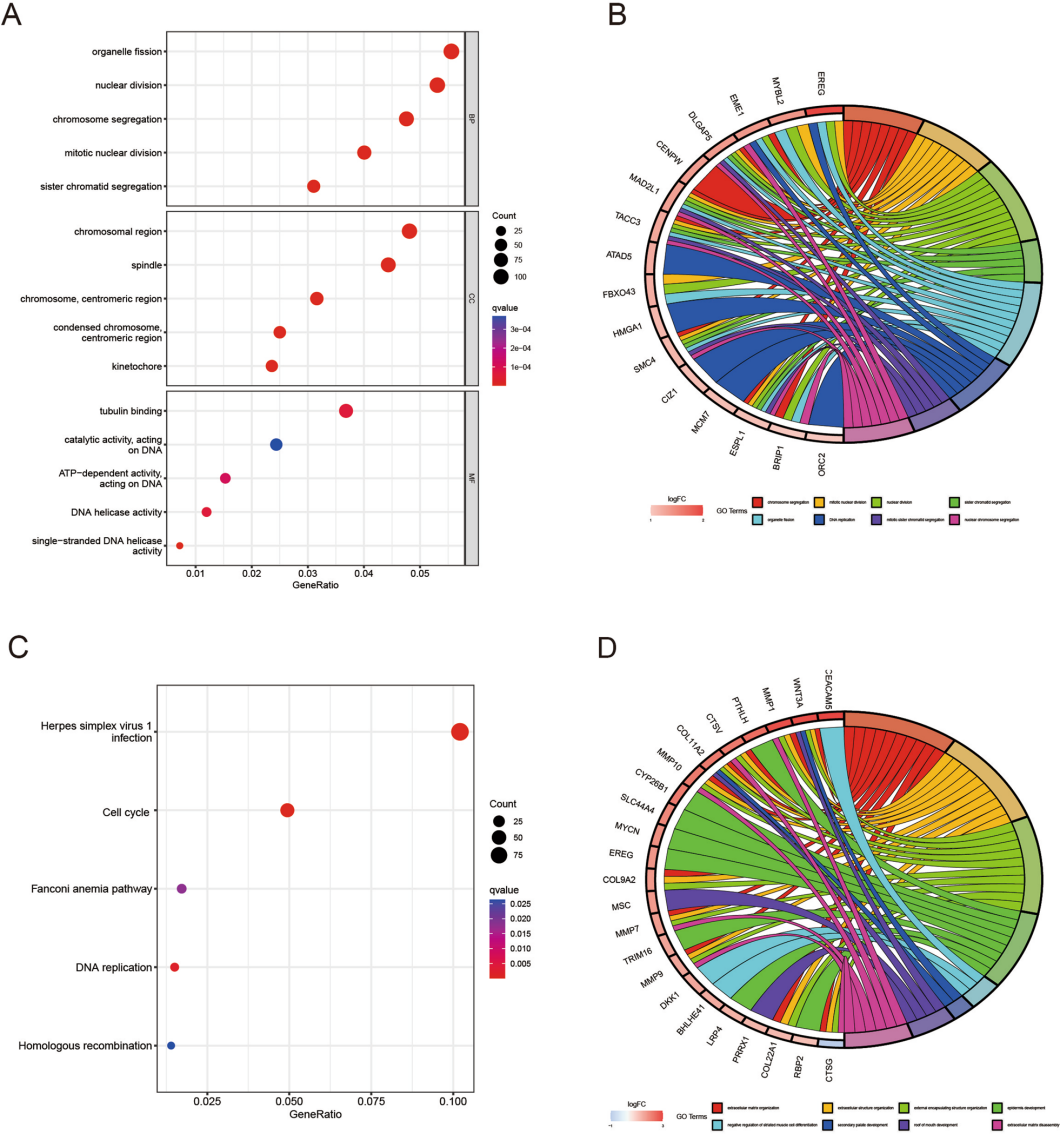
GO and KEGG analysis of differentially expressed genes between the high- and low-risk groups. **A** The bar plot of GO enrichment analysis. The top 5 terms were significantly enriched in GO categories for BP, CC, and MF, respectively. **B** The circos plot of interconnection between GO terms and genes. **C** The bubble plot for KEGG enrichment analysis. **D** The circos plot of interconnection between KEGG terms and genes

To indicate the biological functions and pathways that were associated with the risk score, we obtained the differentially expressed genes between the high- and low-risk groups with a cutoff value of |log2fold change|> 0.5 and false discovery rate (FDR) < 0.05, including 2784 up-regulated genes and 158 down-regulated genes (Additional file 8: Table S1). Then, we performed GO and KEGG enrichment analysis of these DEGs to identify the biological processes correlated with the risk score (Additional file 9: Table S2). GO enrichment analysis involving the BP category indicated that these DEGs are predominantly associated with organelle fission, nuclear division and chromosome segregation (Fig. 5A, B). For the CC category, enriched DEGs were mainly related to chromosomal region and spindle. For the MF category, enriched DEGs were largely related to tubulin binding and catalytic activity on DNA. KEGG pathway analysis showed that Herpes simplex virus 1 infection, Cell cycle and Fanconi anemia pathway were significantly enriched with the DEGs (Fig. 5C, D).

### Assessment of response of high-risk and low-risk patients with LIHC to candidate drugs

To further evaluate the response of LIHC patients to candidate drugs in the high-risk and low-risk groups, we assessed the sensitivity score for each compound for each patient in the high-risk and low-risk groups. We identified 71 compounds with IC50 values that significantly differences between the high-risk and low-risk groups (Additional file 10: Table S3). In addition, using the PubChem website, 2D conformations of the four compounds with the most significant differences in sensitivity score between the high- and low-risk groups were visualized, including Gemcitabine (Additional file 7: Figure S7A), Epothilone.B (Additional file 7: Figure S7B), Embelin (Additional file 7: Figure S7C) and AMG.706 (Additional file 7: Figure S7D).

### Tumour mutation analysis between high-risk and low-risk populations

**Fig. 6:**
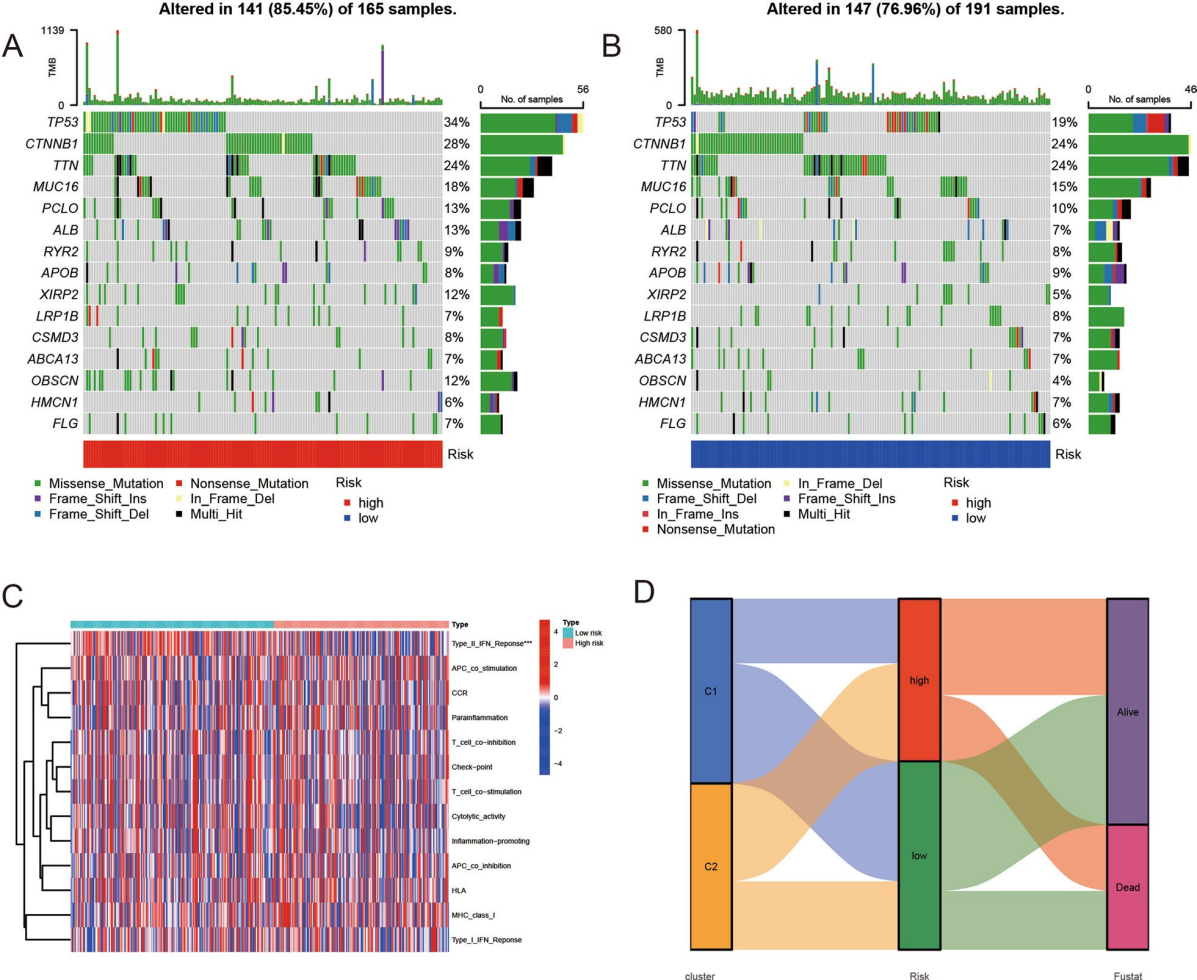
Differences in mutation frequency between high- and low-risk groups. **A**, **B** Comparing the degree of mutation between high and low risk groups, the abscissa represents the sample, and the ordinate represents the mutated gene. **C** A heatmap showing how immune-related functions differ between high- and low-risk groups. **D** Sankey diagram for two subtypes and high and low risk groups

Analysis and visualization of the top 15 genes associated with TMF in LIHC patients were performed using the maftool package in R software. In high-risk populations, TP53 mutation frequency is highest (Fig. 6A). In low-risk populations, CTNNB1 and TTN mutations are most frequent (Fig. 6B). Heatmap of immune-related function analysis showed significant differences in immune function between patients in the high and low risk groups (Fig. 6C). The Sankey showed that patients in the C1 subtype and low-risk groups had a better prognosis (Fig. 6D).

### Identifying LIPT1 as prognosis marker for LIHC

**Fig. 7:**
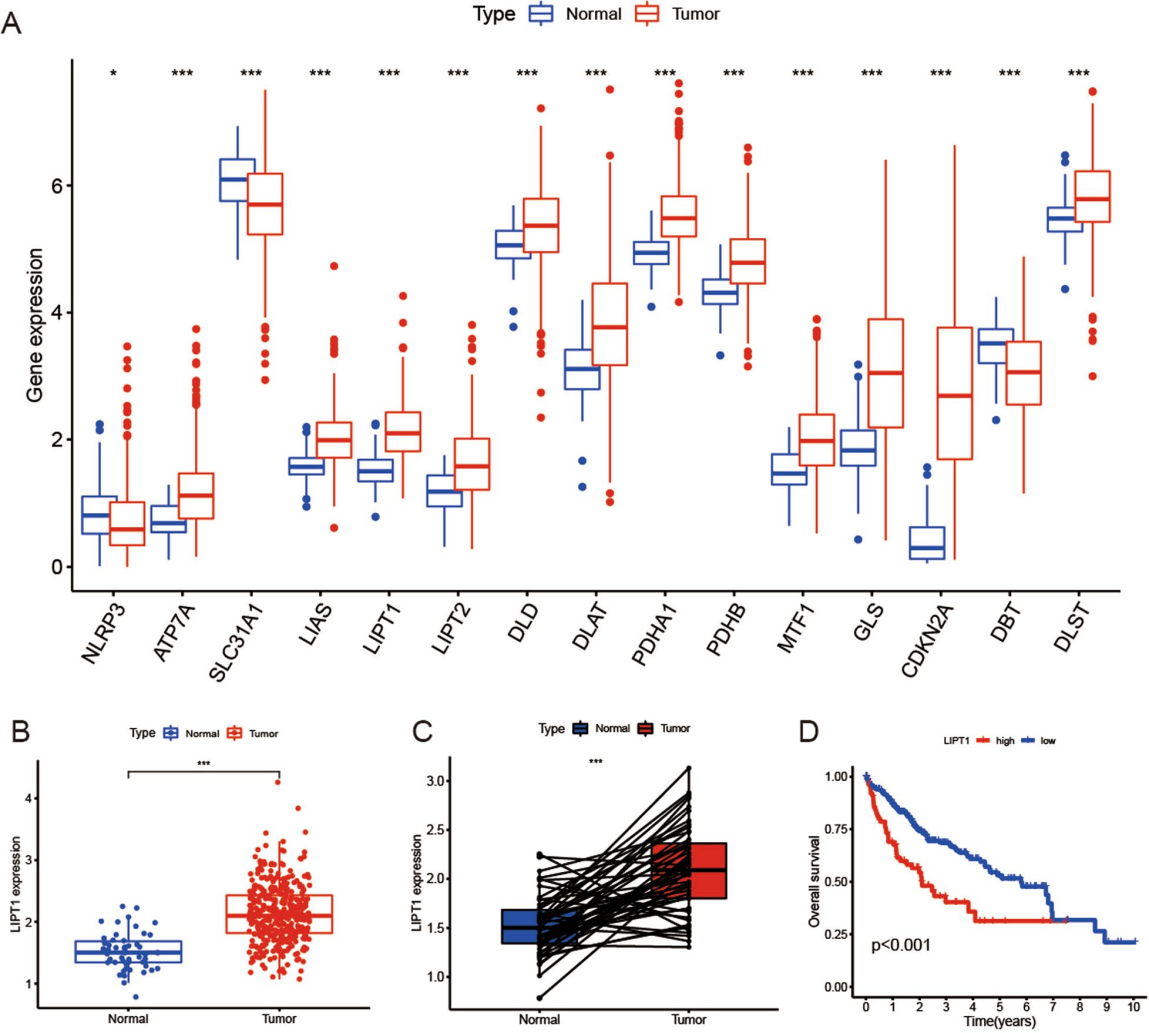
Identifying LIPT1 as prognosis marker for LIHC. **A** Box plots show gene expression values for cuproptosis-related genes. Blue represents normal samples and red represents tumor samples **B** LIPT1 expression levels of normal and breast cancer tissue in TCGA. **C** Relative expression of LIPT1 in hepatocellular carcinoma tissues and adjacent non-cancerous tissues in TCGA database. **D** OS curves for LIHC patients with high and low LIPT1 expression

To identify cuproptosis-related prognosis markers for LIHC, we analyzed the expression levels of cuproptosis-related genes expression in cancer and normal tissues. Box plots showed that 15 genes were differentially expressed between normal and tumor tissue samples (P < 0.05) (Fig. 7A). Among them, 12 genes were highly expressed level in tumor tissues, including ATP7A, LIAS, LIPT1, LIPT2, DLD, DLAT, PDHA1, PDHB, MTF1, GLS, CDKN2A and DLST. And the others are highly expressed level in normal tissues, including NLRP3 and SLC31A1. The scatter plot showed that LIPT1 levels were higher in LIHC tumor samples compared with normal samples (Fig. 7B). LIPT1 was statistically significantly higher expressed in the LIHC tissues when compared with paired adjacent normal tissues in TCGA cohort (P < 0.001) (Fig. 7C). Survival curves showed that patients with low LIPT1 expression level had significantly longer OS compared with with high LIPT1 expression level (Fig. 7D).

### Knockdown of LIPT1 inhibited LIHC cell proliferation and migration

To validate the biological function of LIPT1 in LIHC, we knocked down LIPT1 using two siRNAs in HepG2 and Hep3B cells.

**Fig. 8:**
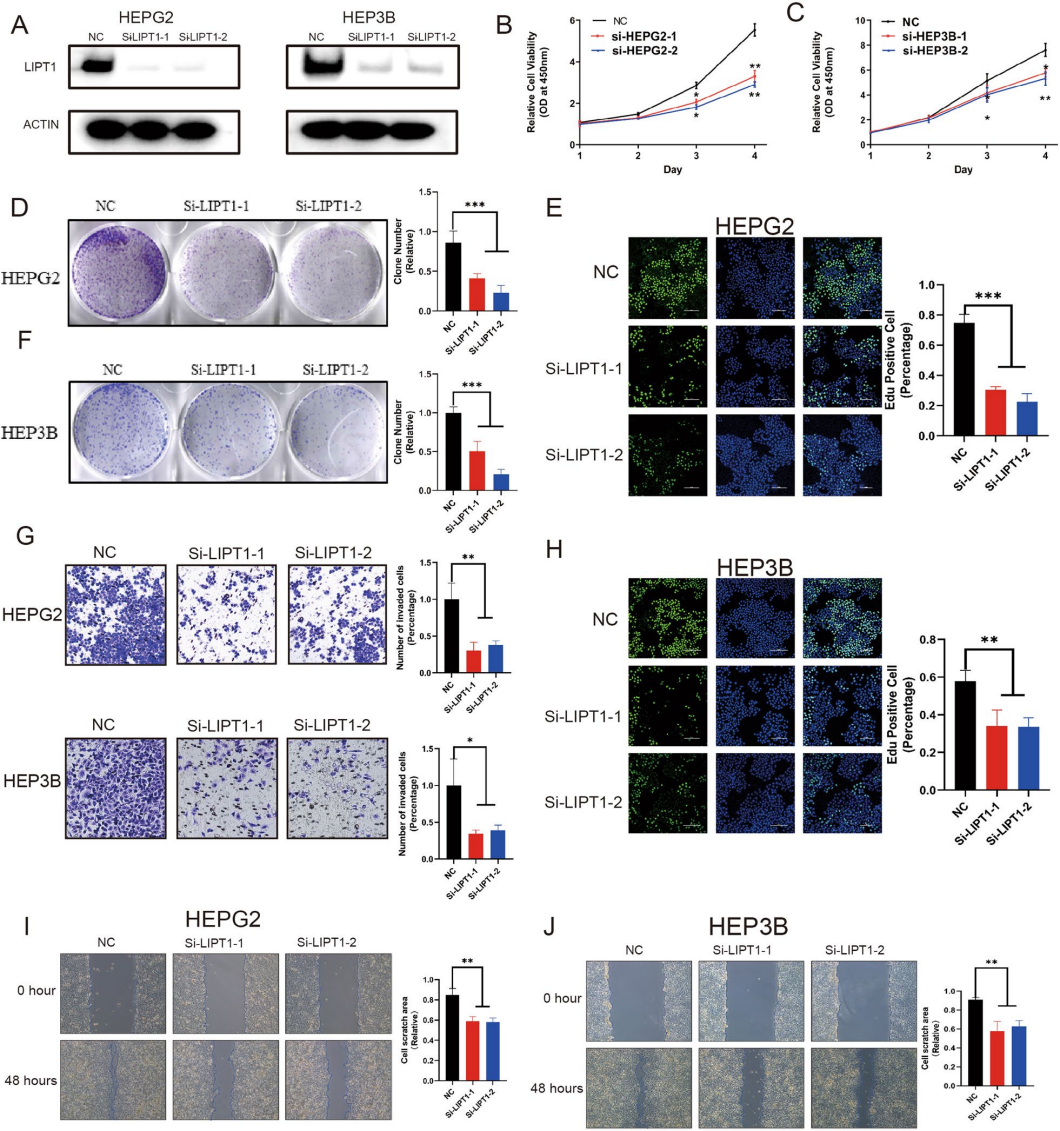
LIPT1 promotes proliferation, invasion and migration of LIHC cells. **A** Western blot to show knockdown efficiency of LIPT1 in HepG2 and Hep3B cells by two independent siRNAs. **B**, **C** Cell proliferation of HepG2 cells **B** or Hep3B cells **C** transfected with control or si-LIPT1 was measured by CCK8. **D**, **F** Colony formation of HepG2 cells or Hep3B cells transfected with control or si-LIPT1 was measured by ImageJ. **E**, **H** Edu assay to show the cell proliferation of control cells comparing to LIPT1 knockdown cells. **G** Transwell assay to show the cell metastasis of control cells compared to LIPT1 knockdown cells. **I**, **J** Wound healing assay to show the cell migration of control cells compared to LIPT1-depleted cells

Western blotting showed that LIPT1 could be effectively silenced by two independent siRNAs (Fig. 8A). The CCK8 assay showed that LIPT1 depletion inhibited cancer cell proliferation in HepG2 and Hep3B cells (P < 0.05, Fig. 8B, C). Knockdown of LIPT1 inhibits cell proliferation and clone formation capability in HepG2 and Hep3B cells lines (Fig. 8D, F). Edu staining results showed that knockdown of LIPT1 significantly decreased LIHC cell proliferation (Fig. 8E, H). The trans-well assay showed that LIPT1 knocking-down inhibited cell invasion capacity in HepG2 and Hep3B cells (Fig. 8G). Subsequently, in the wound-healing assay, we found that LIPT1 depletion inhibited wound closure speed in both HepG2 and Hep3B cells (Fig. 8I, J). These results indicated that LIPT1 could promote hepatocellular carcinoma cell proliferation and migration in vitro. LIPT1 is likely to be a potential target for hepatocellular carcinoma therapy.

## Discussion

A recent study found that accumulation of intracellular copper triggers aggregation of mitochondrial lipoproteins and destabilization of proteins, leading to a unique type of cell death called cuproptosis [11, 12]. Copper plays an important role in cells as a catalytic cofactor for essential enzymes involved in energy conversion, oxygen transport, and regulation of oxidative metabolism in cells [7]. The concentration of copper in cells is regulated by metabolic demands and changes in the cellular environment, and too little or too much can cause significant damage to cells [8]. Imbalance of copper metabolism can seriously affect the development of the central nervous system and have an impact on the normal metabolism of the liver [9]. When the intracellular copper concentration reaches a certain level, copper ions directly bind to the lipidated component of the TCA cycle, resulting in abnormal aggregation of fatty acylated proteins and loss of iron-sulfur cluster proteins, ultimately leading to cell death mediated by proteotoxic stress responses [10]. Cuprotosis is closely associated with cancer progression and is expected to be a novel therapeutic target to specifically induce cancer cell death [13, 14]. However, the prognostic model of LIHC based on cuproptosis-related genes has not been reported.

In this study, we developed a prognostic risk model based on three cuproptosis-related genes by performing LASSO cox regression and multivariate cox regression analysis. Both in the training and in the test sets, the OS of LIHC patients in the low-risk group was significantly longer than that in the high-risk group. The Kaplan–Meier curve and the ROC curve were performed to estimate the sensitivity and specificity of the prognostic signature. By performing NMF cluster, we identified two molecular subtypes of LIHC (C1 and C2), with C1 subtype having significantly longer OS and PFS than C2 subtype. We then performed drug sensitivity analysis that may provide a new reference for selection of treatment strategies for LIHC patients. Finally, we validated the function of LIPT1 in LIHC by knocking down its expression level, LIPT1 may provide a potential therapeutic target. cancer, according to previous research.

These three genes play an important role in the development and metastasis of many types of cancer.

The three genes (GCSH, LIPT1, CDKN2A) that we used to construct cuproptosis-related prognostic models played important roles in the progression of various types of cancer. Intracellular GCSH content is a critical factor in determining cellular metabolic status and viability, including tumorigenesis, and it has been shown that GCSH is an effective tumor marker in breast cancer [24]. LIPT1 is the gene encoding fatty acyltransferase 1, a key factor in regulating lipoic acid (LA) transport [25]. LA plays an important role in tricarboxylic acid cycle and mitochondrial metabolism in cancer cells [26, 27]. CDKN2A is the gene encoding the cell cycle inhibitor p16^CDKN2A^, and the expression level of p16^CDKN2A^ is closely related to colorectal cancer invasion or metastatic potential [28, 29]. In addition, it has been shown that CDKN2A is a new marker of poor prognosis in patients with hepatocellular carcinoma [30], which is consistent with our study.

KEGG pathway analysis showed that differential expressed genes between high and low risk groups were significantly enriched for cell cycle signaling pathways. Dysregulation of the cell cycle underlies aberrant cell proliferation in cancer [31, 32]. CDKN2A is the gene encoding the cyclin inhibitor p16 protein, which prevents abnormal cell growth and proliferation by binding to a complex of cyclin-dependent kinases 4 and 6 and cyclin D [29]. Abnormal expression levels of cellular CDKN2A may lead to enhanced tumorigenesis and metastasis [33].

Subsequently, we identified 71 compounds with IC50 values that significantly differences between the high-risk and low-risk groups. Gemcitabine is a pyrimidine nucleoside antimetabolite that has been approved for the treatment of non-small cell lung cancer, pancreatic cancer, bladder cancer, and breast cancer [34, 35]. Ebomycin is a macrolide with good anticancer activity and its mechanism of action is similar to paclitaxel. Meanwhile, epothilone B is also highly active against cancer cells resistant to paclitaxel and other anticancer drugs [36]. Embelin is a naturally occurring benzoquinone compound that has been shown to have many biological properties associated with cancer prevention and treatment [37]. Embelin can induce apoptosis by modulating NF-κB, p53, PI3K/AKT, and STAT3 signaling pathways [37, 38]. In addition, it has been shown that Embelin induces autophagy in cancer cells in ovarian cancer [39]. AMG706 is a multikinase inhibitor that has been experimentally demonstrated to have antiproliferative, antiangiogenic, and apoptotic effects on colorectal cancer cells [40]. However, further studies are still needed to evaluate the effectiveness of these drugs in the treatment of LIHC. Our results may provide new insights into the treatment of patients with LIHC.

Oxidative stress (OS), a state characterized by an imbalance between pro-oxidant molecules including reactive oxygen and nitrogen species, and antioxidant defenses, is associated with the hepatocarcinogenesis [41–45]. Therefore, antioxidant therapy may potentially be effective for suppressing progression and metastasis of hepatocellular carcinoma. Recent studies have indicated that antioxidants may be potential candidates for the treatment of HCC since the main treatment includes surgical removal and liver transplantation [46]. Resveratrol is a polyphenolic compound naturally found in several dietary sources, such as grapes, berries, peanuts, and red wine, which is well known as the compound to reduce the incidence of heart disease [47]. By decreasing the p-ERK expression and increasing p-JNK expression, Resveratrol significantly dramatically inhibited hepatocarcinoma cell viability and induced apoptosis in vitro and in vitro [48]. Similarly, Quercetin inhibits hepatocellular carcinoma progression by down-regulation of the activation of JAK2 and STAT3. Gallic acid show strong antitumor potential in the treatment of cellular hepatocarcinoma in vivo and in vitro [49, 50]. Nevertheless, clinical trials have not yet been conducted to confirm their effectiveness in humans. Although antioxidants may be potentially appropriate in patients with hepatocellular carcinoma, there is still an urgent need for novel and improved drug identification. Citalopram, anti-depressant agents, have been demonstrated it has the promising properties of anti-cancer effect in liver cancer, bladder cancer, breast cancer, colorectal carcinoma and neuroblastoma [51–54]. Citalopram exert cytotoxic effects on liver cancer cells by through cytochrome c release and ROS-dependent activation of NFκB [53].

We selected two cell lines, HepG2 and Hep3B, for further experiments on LIPT1. Western blotting showed that LIPT1 could be effectively silenced by two independent siRNAs. The CCK8 assay showed that LIPT1 depletion inhibited cancer cell proliferation in HepG2 and Hep3B cells. Knockdown of LIPT1 inhibits cell proliferation and clone formation capability in HepG2 and Hep3B cells lines. Edu staining results showed that knockdown of LIPT1 significantly decreased LIHC cell proliferation. The trans-well assay showed that LIPT1 knocking-down inhibited cell invasion capacity in HepG2 and Hep3B cells. Subsequently, in the wound-healing assay, we found that LIPT1 depletion inhibited wound closure speed in both HepG2 and Hep3B cells. Our results suggest that LIPT1 can promote the proliferation, invasion and migration of hepatocellular carcinoma. It turns out that LIPT1 is likely to be a very important potential target for the treatment of hepatocellular carcinoma.

The circulating renin-angiotensin system (RAS) is mainly known for its vital function in maintaining cardiovascular homeostasis, electrolyte balance and kidney function [55]. Angiotensin I (Ang I), angiotensin II (Ang II), angiotensin-converting enzyme (ACE), angiotensin-converting enzyme 2 (ACE2), and angiotensin (Ang) are considered essential elements of the RAS system [56, 57]. Interestingly, previous studies suggest that RAS is involved in the formation and development of LIHC [56, 58]. The Ang II / Ang II type 1 receptor (AT1R) axis can promote tumor progression and metastasis, while the ACE2/Ang- (1 − 7)/MasR axis plays an opposite role [55, 59, 60]. Accordingly, some studies have indicated that AT1R is highly expressed in LIHC samples [61, 62]. Ang II is shown to increase vascular endothelial growth factor (VEGF) and promote tumor-associated, VEGF-induced, ischemia-induced angiogenesis in liver cancer [63–65]. What’s more, some studies support that use of inhibitors of RAS is associated with better prognosis in patients with hepatocellular carcinoma [66]. Moreover, many studies have demonstrated that the upregulation of local RAS in the liver is associated liver fibrosis, which eventually develops into cirrhosis or even hepatocellular carcinoma [67–70]. In addition, renin angiotensin system inhibitor therapy results in a reduction in liver fibrosis score and liver fibrosis area in patients with liver fibrosis [71].These results indicate that targeting RAS may be a promising approach for the treatment of LIHC.

LIPT1 protein transfers a lipoyl moiety from lipoyl-adenylate to both glycine cleavage system protein H (GCSH) and to the 2-oxoacid dehydrogenase E2 subunits, which is involved in the metabolism of lipoic acid [10, 72]. Mutations in the LIPT1 gene were indicated to cause some genetic disorders, such as a Leigh disease with secondary deficiency for pyruvate and alpha-ketoglutarate dehydrogenase and a fatal disease related to a specific lipoylation defect of the 2-ketoacid dehydrogenase complexes [73, 74]. It has been found that LIPT1 is a favorable prognosis in patients with urothelial cancer or melanoma [75, 76]. In this study, we found that LIPT1 was upregulated in LIHC and an independent prognostic factor for poor prognosis of LIHC, which is different from previous study. Our results clearly indicated that genes played different role in different tumor types, which has been proved in previous study [77]. The point that the same gene can function in completely opposite ways in different cell types is crucial for understanding cellular fate decisions in cancer. The mechanisms underlying the regulation of LIPT1 on LIHC is need to be explored in the future. Our results provide a novel target gene for the treatment of LIHC.

However, there are some limitations to the study. Although LIPT1 can significantly affect the proliferation, invasion and metastasis of hepatocellular carcinoma, the specific mechanism is still unclear. We intend to explore the mechanism underlying the regulation of LIPT1 on LIHC both in vivo and in vitro.

## Conclusion

In this study, we developed a prognostic model based on GCSH, LIPT1 and CDKN2A genes, which effectively predicted the prognosis of LIHC patients. Screening of four potential drugs that may be effective in treating patients with hepatocellular carcinoma. LIPT1 plays an important role in hepatocellular carcinoma, which affects proliferation, invasion, and migration of this type of cancer. LIPT1 may be a very important target in the treatment of hepatocellular carcinoma.

## Supplementary Information

**Additional file 1: Figure S1.** Identification of the cuproptosis-related gene by Lasso cox regression analysis in LIHC. **A** Partial likelihood deviance with changing of log (λ) plotted through LASSO Cox regression in 10-fold cross-validations. **B** Coefficients with changing of log (λ) plotted through LASSO Cox regression in 10-fold cross-validations. **C** Forest plot for multivariate Cox regression analysis of cuproptosis-related genes.

**Additional file 2: Figure S2.** An independent prognostic analysis of clinical parameters and risk scores. **A** The univariate Cox regression analysis of the associations between the risk scores and clinical parameters and the OS of patients in training set. **B** The multivariate Cox regression analysis of the associations between the risk scores and clinical parameters and the OS of patients in training set. **C** The univariate Cox regression analysis of the associations between the risk scores and clinical parameters and the OS of patients in test set. **D** The multivariate Cox regression analysis of the associations between the risk scores and clinical parameters and the OS of patients in test set.

**Additional file 3: Figure S3.** Establishment of the nomogram to predict overall survival of LIHC patients based on TCGA cohort. **A** The nomogram for predicting survival proportion of patients in 1-, 3-, and 5 year. **B**–**D** The calibration plots for predicting patient survival at 1-, 3- and 5 years.

**Additional file4: Figure S4.** The immune infiltration of 22 immune cell types in high and low risk patients with LIHC. **A** The comparison of the proportion of immune cells infiltrating in high- and low-risk patients. **B** The heatmaps plot of immune cell infiltrating in high- and low-risk groups. **C** The violin plot of immune cell infiltrating in high- and low-risk patients.

**Additional file 5: Figure S5.** An analysis of immune cells to predict the survival of LIHC patients. An example of Kaplan-Meier curves for high- and low-risk subjects in different groups, including **A** Plasma cells and **B** T cells CD8.

**Additional file 6: Figure S6.** Correlation of immune checkpoints and risk score. **A** Box plots of immune checkpoint molecule expression between high-risk and low-risk groups. **B** An analysis of the Spearman correlation between immune checkpoints and risk scores. Blue represents a negative correlation, while red represents a positive correlation.

**Additional file 7: Figure S7.** Drug sensitivity correlated with high-and low-risk patients in liver hepatocellular carcinoma. **A** IC 50 value of Gemcitabine in high-and low-risk patients with LIHC. **B** IC 50 value of Epothilone.B in high-and low-risk patients with LIHC. **C** IC 50 value of Embelin in high-and low-risk patients with LIHC. **D** IC 50 value of AMG.706 in high-and low-risk patients with LIHC.

**Additional file 8: Table S1.** Differentially expressed genes between high and low risk groups.

**Additional file 9: Table S2.** Gene Ontology (GO) term enrichment and Kyoto Encyclopedia of Genes and Genomes (KEGG) pathway analysis.

**Additional file 10: Table S3.** Drugs with significant differences in IC50 values between high-risk and low-risk groups.

## Data Availability

Any data and R script in this study can be obtained from the corresponding author upon reasonable request. The final manuscript was read and approved by all authors. In this study, publicly available datasets were analyzed. These are available on The Cancer Genome Atlas (https://portal.gdc.cancer.gov/) and GEO (https://www.ncbi.nlm.nih.gov/).
