# Correction: System analysis based on the cuproptosis-related genes identifies LIPT1 as a novel therapy target for liver hepatocellular carcinoma

**DOI:** 10.1186/s12967-025-07008-x

**Published:** 2026-05-08

**Authors:** Cheng Yan, Yandie Niu, Liukai Ma, Lifang Tian, Jiahao Ma

**Affiliations:** School of Pharmacy, Key Laboratory of Nano‑Carbon Modified Film, Technology of Henan Province, Diagnostic Laboratory of Animal Diseases, Xinxiang University, Xinxiang, Henan China

**Correction: Journal of Translational Medicine (2022) 20:452.**

10.1186/s12967-022-03630-1

Following publication of the original article [1], the authors reported inadvertent image misuses in Fig. 8 as well as errors in the Figure Legend.

The incorrect version of Fig. 8 and Legend was:

**Fig. 8:**
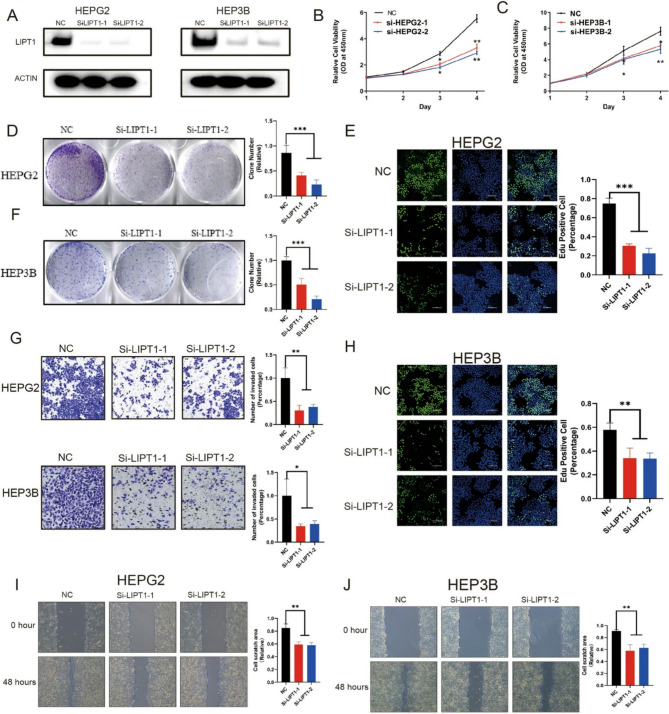
LIPT1 promotes proliferation, invasion and migration of LIHC cells. **A** Western blot to show knockdown efficiency of LIPT1 in HepG2 and Hep3B cells by two independent siRNAs. **B**, **C** Cell proliferation of HepG2 cells **B** or Hep3B cells **C** transfected with control or si-LIPT1 was measured by CCK8. **D**, **F** Colony formation of HepG2 cells or Hep3B cells transfected with control or si-LIPT1 was measured by Image **J**. **E**, **H** Edu assay to show the cell proliferation of control cells comparing to LIPT1 knockdown cells. **G** Transwell assay to show the cell metastasis of control cells compared to LIPT1 knockdown cells. **I**, **J** Wound healing assay to show the cell migration of control cells compared to LIPT1-depleted cells

The correct Fig. 8 and Legend are:

**Fig. 8:**
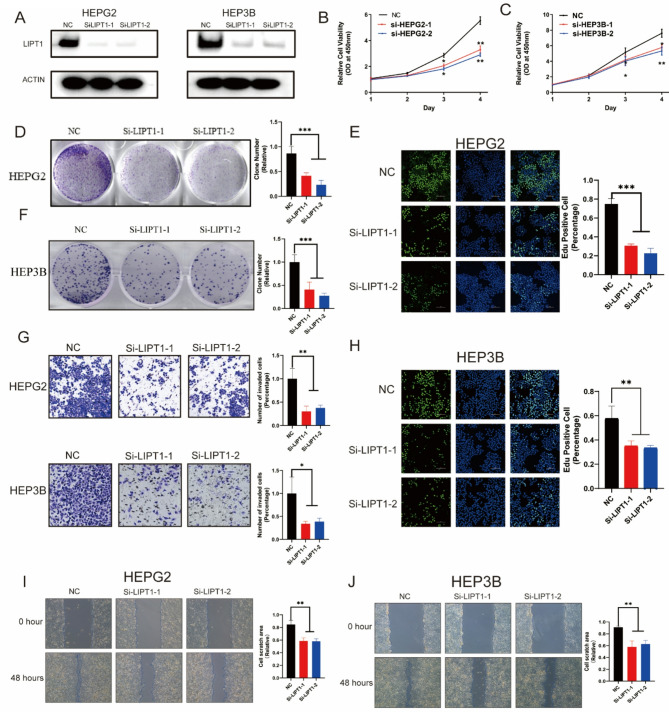
LIPT1 promotes proliferation, invasion, and migration of LIHC cells. **A** Western blot analysis showing the knockdown efficiency of LIPT1 in HepG2 and Hep3B cells using two independent siRNAs. **B**, **C** CCK-8 assay showing cell proliferation of HepG2 (**B**) and Hep3B (**C**) cells transfected with control siRNA or si-LIPT1. **D**, **F** Colony formation assay of HepG2 (**D**) and Hep3B (**F**) cells transfected with control or si-LIPT1. **E**, **H** EdU incorporation assay showing proliferative activity in control versus LIPT1-knockdown HepG2 (**E**) and Hep3B (**H**) cells. **G** Transwell assay assessing the invasive capacity of control and LIPT1-knockdown cells. **I**, **J** Wound healing assay showing the migratory ability of control and LIPT1-knockdown cells in HepG2 (**I**) and Hep3B (**J**) lines

The original article [1] has been updated.

## References

[1] Yan C, Niu Y, Ma L, et al. System analysis based on the cuproptosis-related genes identifies LIPT1 as a novel therapy target for liver hepatocellular carcinoma. J Transl Med. 2022;20:452. 10.1186/s12967-022-03630-1. 36195876 10.1186/s12967-022-03630-1 PMC9531858

