# Supplementary figures and images for: System analysis based on the cuproptosis-related genes identifies LIPT1 as a novel therapy target for liver hepatocellular carcinoma
Source: J Transl Med. 2022 Oct 4;20:452. doi: 10.1186/s12967-022-03630-1 (PMC9531858; doi:10.1186/s12967-022-03630-1)

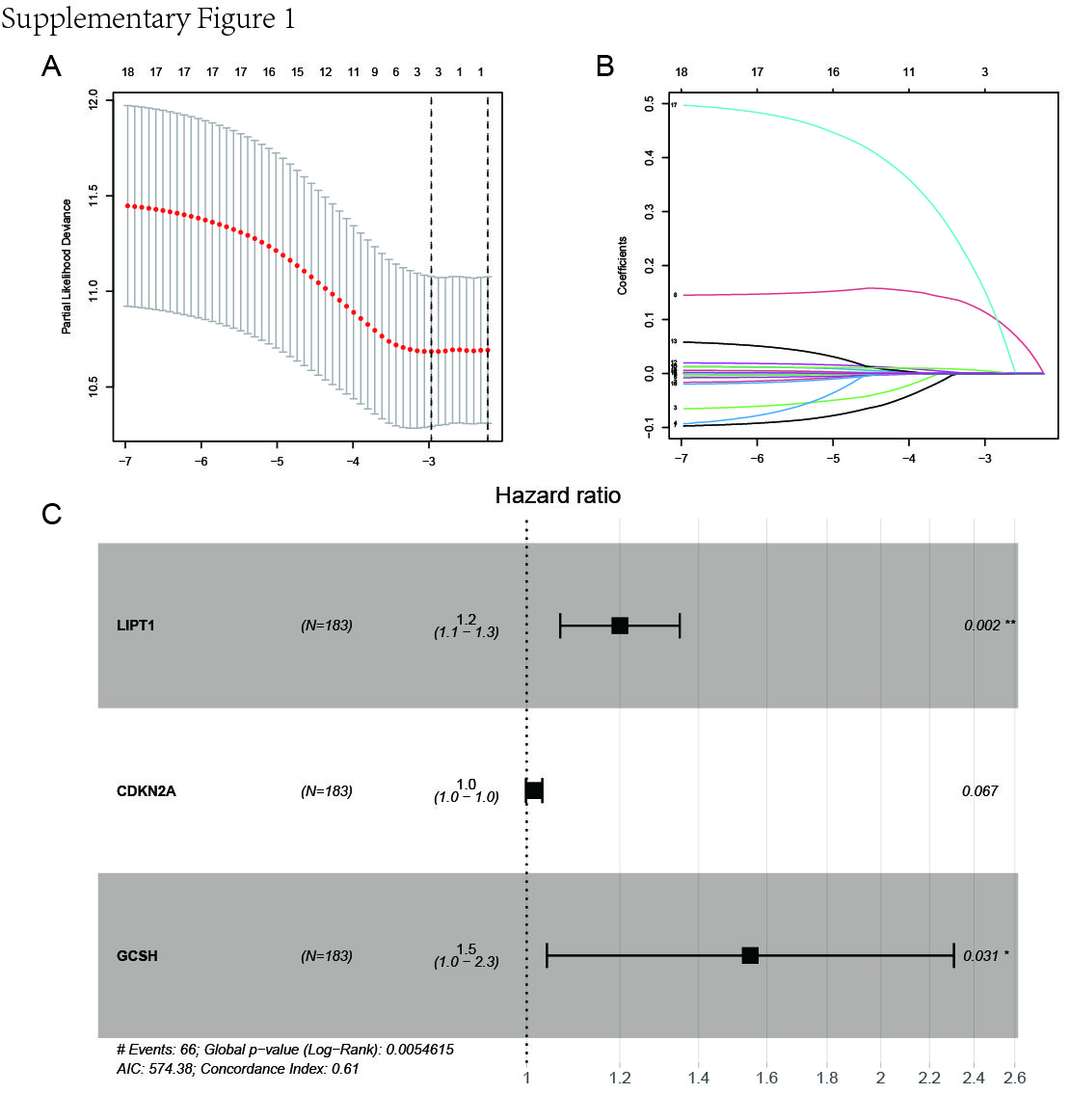

Supplement: Supplementary file 1 — Additional file 1: Figure S1. Identification of the cuproptosis-related gene by Lasso cox regression analysis in LIHC. A Partial likelihood deviance with changing of log (λ) plotted through LASSO Cox regression in 10-fold cross-validations. B Coefficients with changing of log (λ) plotted through LASSO Cox regression in 10-fold cross-validations. C Forest plot for multivariate Cox regression analysis of cuproptosis-related genes. [file 12967_2022_3630_MOESM1_ESM.jpg]

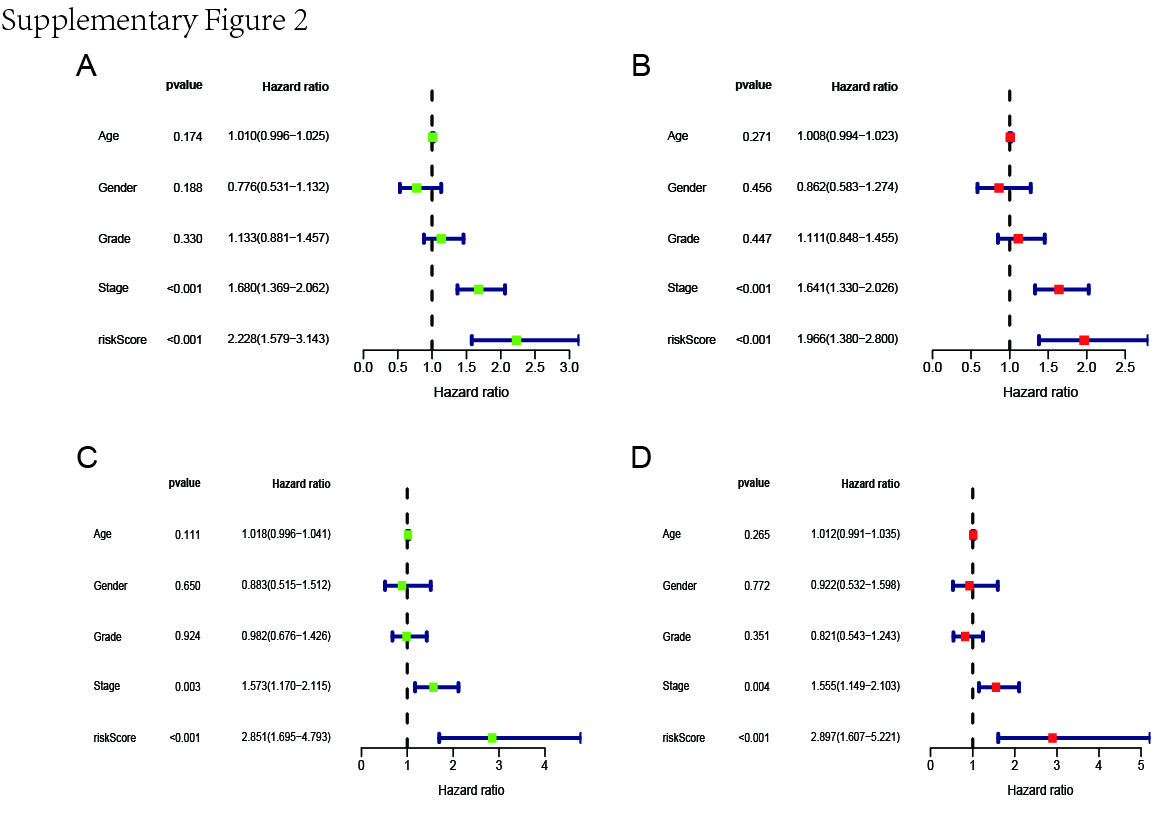

Supplement: Supplementary file 2 — Additional file 2: Figure S2. An independent prognostic analysis of clinical parameters and risk scores. A The univariate Cox regression analysis of the associations between the risk scores and clinical parameters and the OS of patients in training set. B The multivariate Cox regression analysis of the associations between the risk scores and clinical parameters and the OS of patients in training set. C The univariate Cox regression analysis of the associations between the risk scores and clinical parameters and the OS of patients in test set. D The multivariate Cox regression analysis of the associations between the risk scores and clinical parameters and the OS of patients in test set. [file 12967_2022_3630_MOESM2_ESM.jpg]

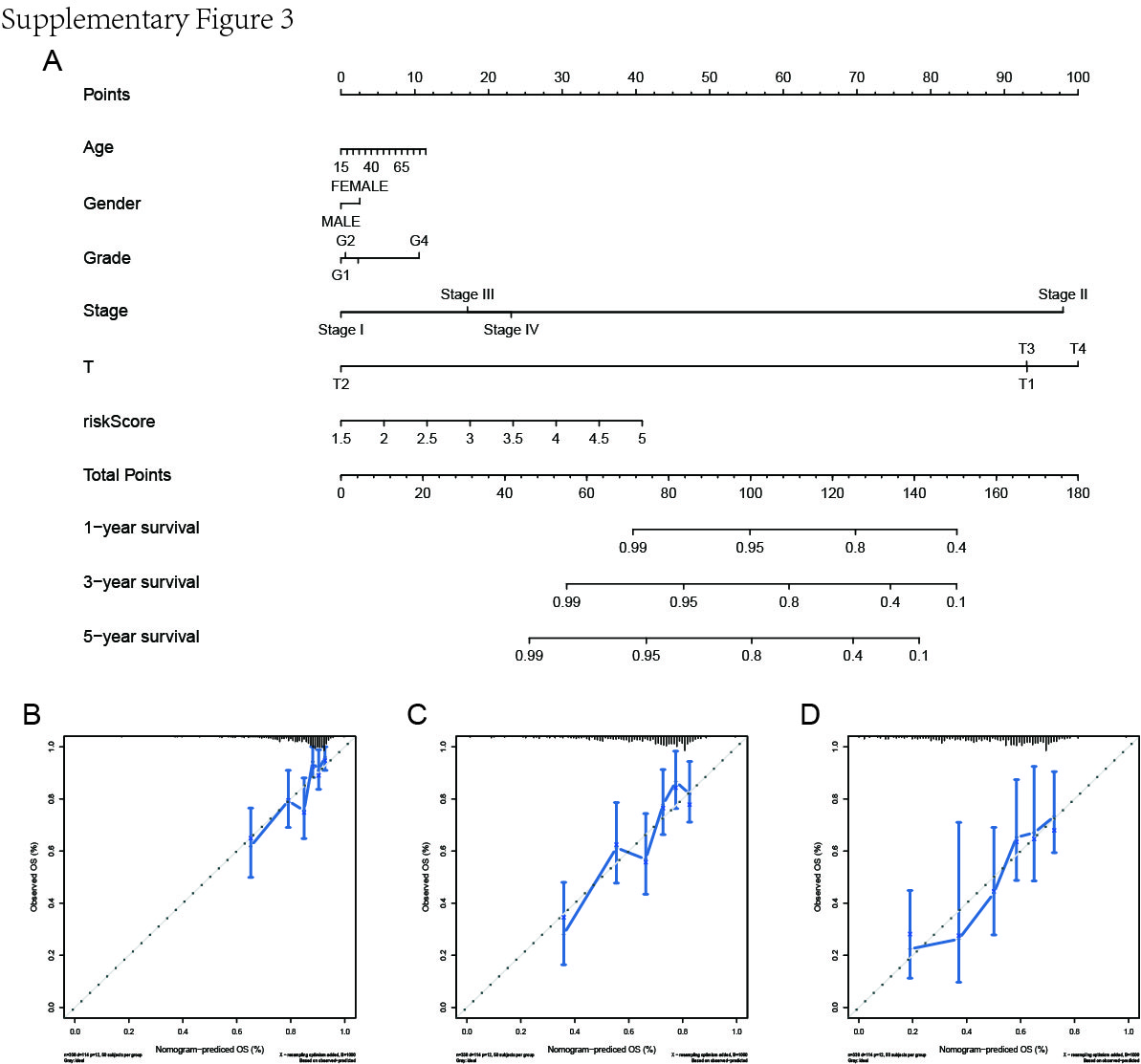

Supplement: Supplementary file 3 — Additional file 3: Figure S3. Establishment of the nomogram to predict overall survival of LIHC patients based on TCGA cohort. A The nomogram for predicting survival proportion of patients in 1-, 3-, and 5 year. B–D The calibration plots for predicting patient survival at 1-, 3- and 5 years. [file 12967_2022_3630_MOESM3_ESM.jpg]

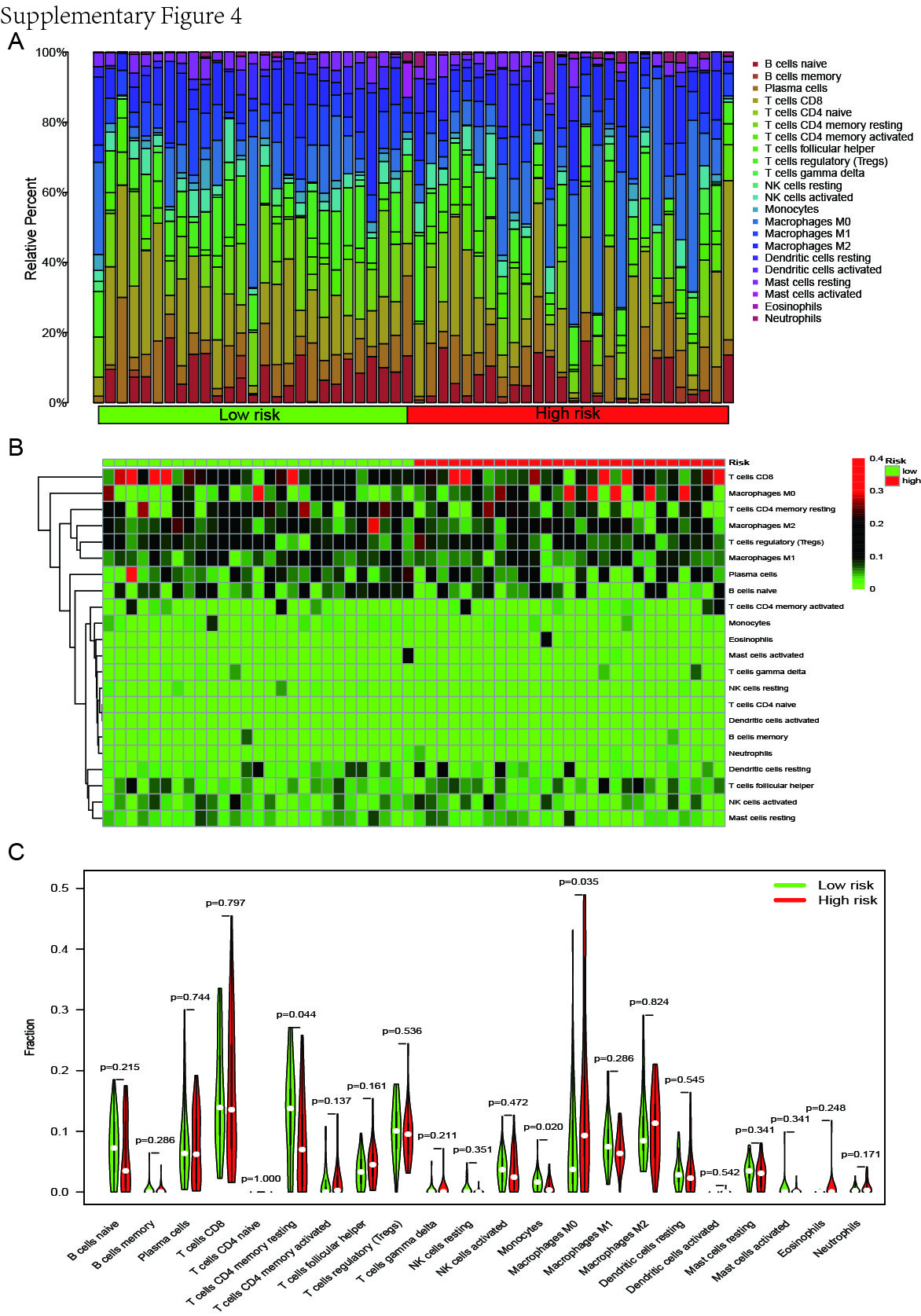

Supplement: Supplementary file 4 — Additional file4: Figure S4. The immune infiltration of 22 immune cell types in high and low risk patients with LIHC. A The comparison of the proportion of immune cells infiltrating in high- and low-risk patients. B The heatmaps plot of immune cell infiltrating in high- and low-risk groups. C The violin plot of immune cell infiltrating in high- and low-risk patients. [file 12967_2022_3630_MOESM4_ESM.jpg]

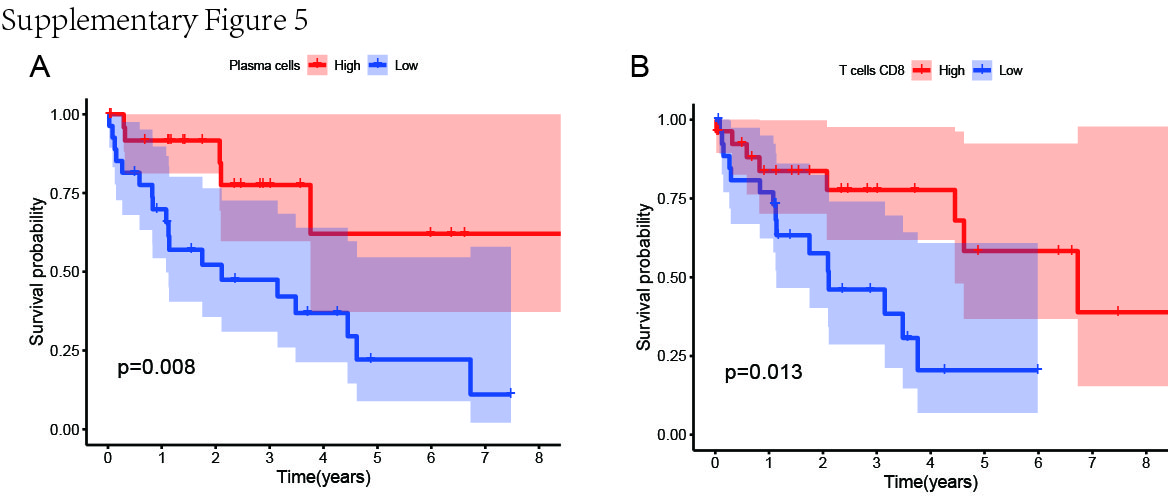

Supplement: Supplementary file 5 — Additional file 5: Figure S5. An analysis of immune cells to predict the survival of LIHC patients. An example of Kaplan-Meier curves for high- and low-risk subjects in different groups, including A Plasma cells and B T cells CD8. [file 12967_2022_3630_MOESM5_ESM.jpg]

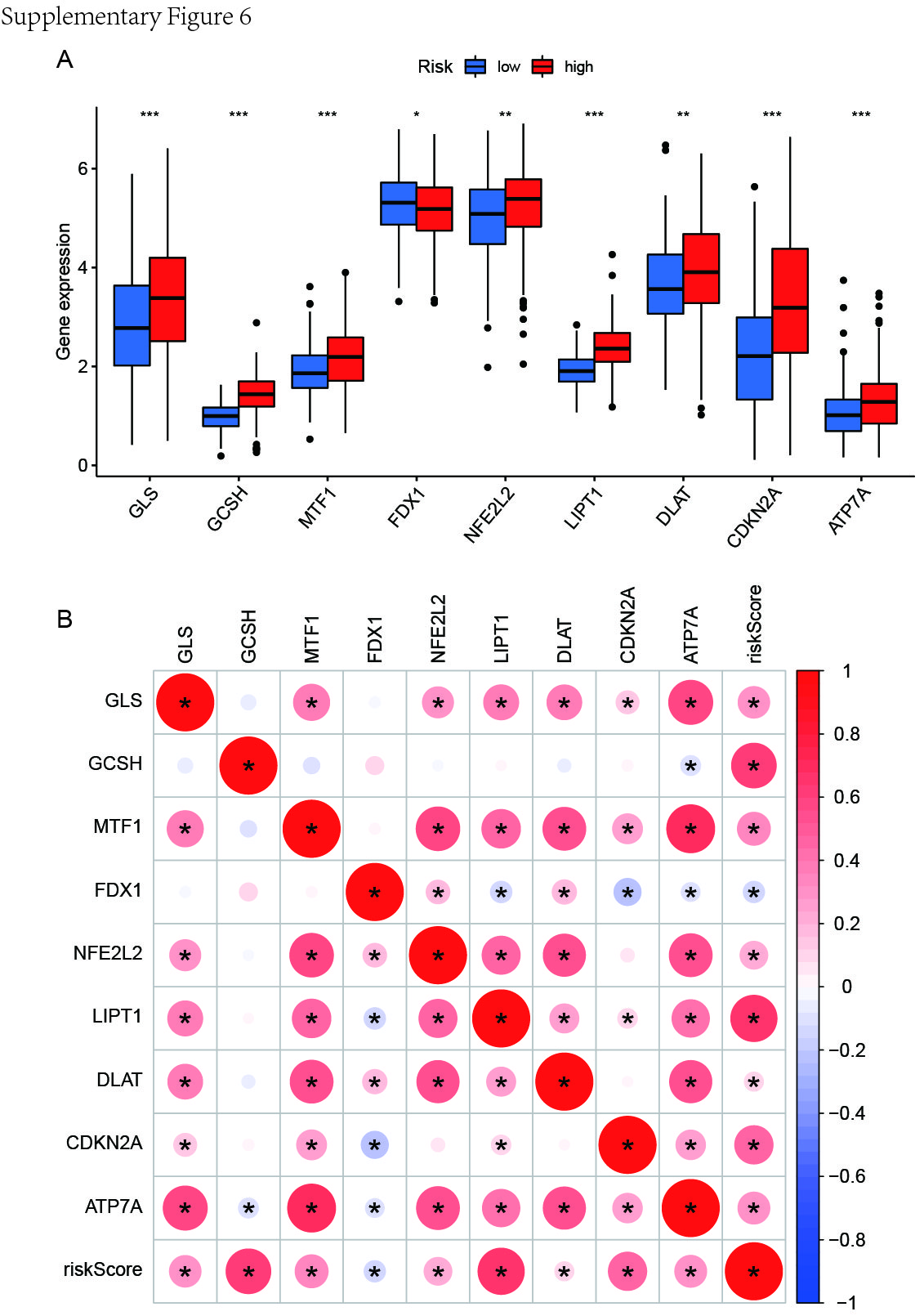

Supplement: Supplementary file 6 — Additional file 6: Figure S6. Correlation of immune checkpoints and risk score. A Box plots of immune checkpoint molecule expression between high-risk and low-risk groups. B An analysis of the Spearman correlation between immune checkpoints and risk scores. Blue represents a negative correlation, while red represents a positive correlation. [file 12967_2022_3630_MOESM6_ESM.jpg]

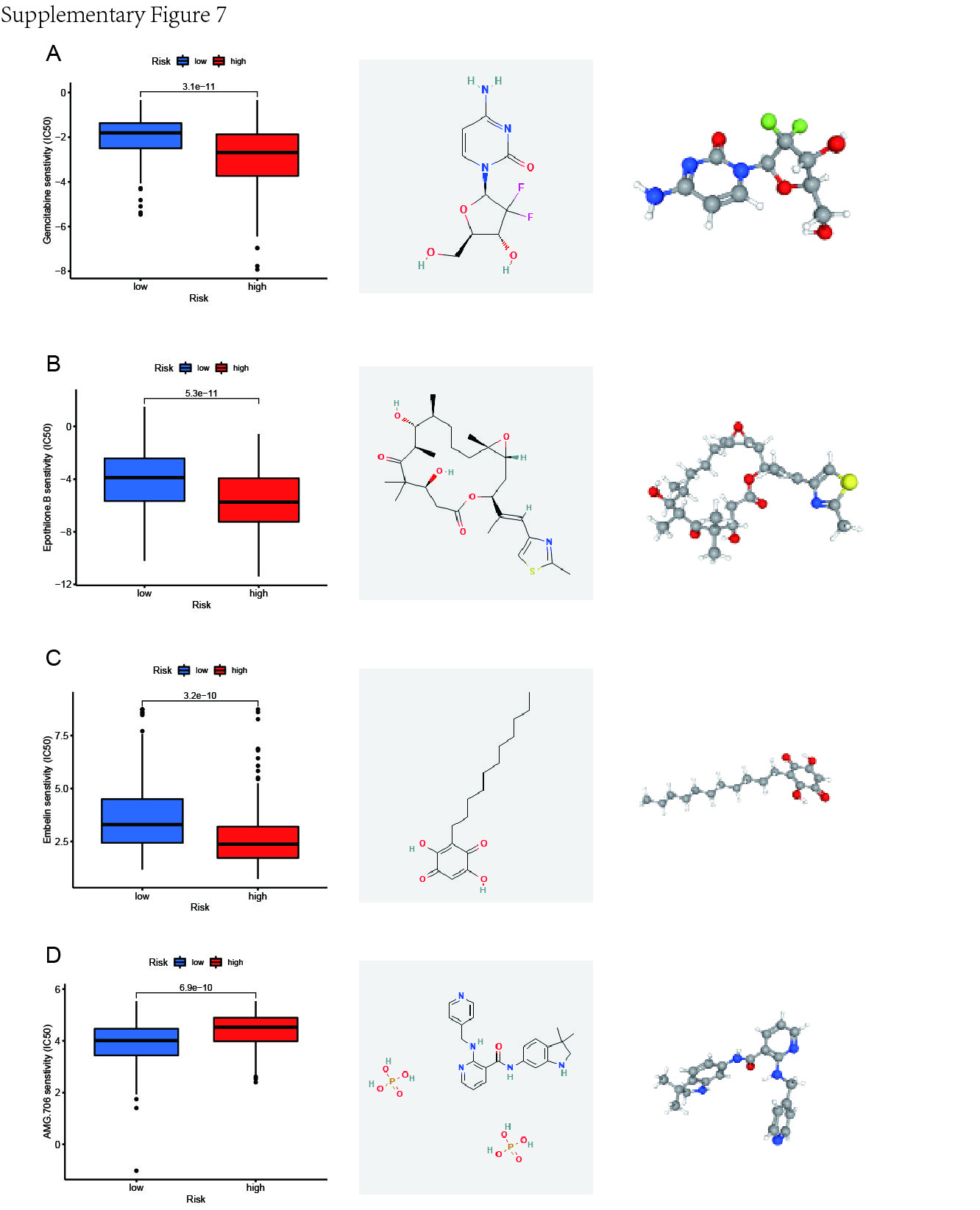

Supplement: Supplementary file 7 — Additional file 7: Figure S7. Drug sensitivity correlated with high-and low-risk patients in liver hepatocellular carcinoma. A IC 50 value of Gemcitabine in high-and low-risk patients with LIHC. B IC 50 value of Epothilone.B in high-and low-risk patients with LIHC. C IC 50 value of Embelin in high-and low-risk patients with LIHC. D IC 50 value of AMG.706 in high-and low-risk patients with LIHC. [file 12967_2022_3630_MOESM7_ESM.jpg]
